# Efficient Exciplex-Based Deep-Blue Organic Light-Emitting Diodes Employing a Bis(4-fluorophenyl)amine-Substituted Heptazine Acceptor

**DOI:** 10.3390/molecules26185568

**Published:** 2021-09-13

**Authors:** Jie Li, Heqi Gong, Jincheng Zhang, Hui Liu, Li Tao, Yanqing Wang, Qiang Guo

**Affiliations:** 1 College of Optoelectronic Engineering, Chengdu University of Information Technology, Chengdu 610225, China; lijie@cuit.edu.cn (J.L.); 3201105022@stu.cuit.edu.cn (H.G.); 3201105004@stu.cuit.edu.cn (J.Z.); liuhui@cuit.edu.cn (H.L.); taoli@cuit.edu.cn (L.T.); 2 College of Polymer Science and Engineering, Sichuan University, Chengdu 610065, China; yanqingwang@scu.edu.cn

**Keywords:** exciplex, deep-blue, organic light-emitting diode, thermally activated delayed fluorescence, heptazine

## Abstract

The realization of a deep-blue-emitting exciplex system is a herculean task in the field of organic light-emitting diodes (OLEDs) on account of a large red-shifted and broadened exciplex emission spectrum in comparison to those of the corresponding single compounds. Herein, 2,5,8-tris(di(4-fluorophenyl)amine)-1,3,4,6,7,9,9b-heptaazaphenalene (HAP-3FDPA) was designed as an electron acceptor by integrating three bis(4-fluorophenyl)amine groups into a heptazine core, while 1,3-di(9*H*-carbazol-9-yl)benzene (mCP) possessing two electron-donating carbazole moieties was chosen as the electron donor. Excitingly, the exciplex system of 8 *wt*% HAP-3FDPA:mCP exhibited deep-blue emission and a high photoluminescence quantum yield of 53.2%. More importantly, an OLED containing this exciplex system as an emitting layer showed deep-blue emission with Commission Internationale de l’Eclairage coordinates of (0.16, 0.12), a peak luminance of 15,148 cd m^−2^, and a rather high maximum external quantum efficiency of 10.2% along with a low roll-off. This study not only reports an efficient exciplex-based deep-blue emitter but also presents a feasible pathway to construct highly efficient deep-blue OLEDs based on exciplex systems.

## 1. Introduction

Organic light-emitting diodes (OLEDs) based on thermally activated delayed fluorescent (TADF) emitters have obtained considerable progress over the last decade [1,2,3,4,5]. As the third-generation organic light-emitting materials, TADF emitters possessing small singlet-triplet energy splitting (∆*E*_ST_) between the lowest excited singlet state (S_1_) and the lowest excited triplet state (T_1_) can harvest both singlet and triplet excitons by efficient up-conversion from T_1_ to S_1_ through a reverse intersystem crossing (RISC) process [6,7,8]. To realize a small ∆*E*_ST_, effective separation of electron densities of the highest occupied molecular orbital (HOMO) and the lowest unoccupied molecular orbital (LUMO) is indispensable because the Δ*E*_ST_ is proportional to the exchange integrals between the wave functions of the HOMO and LUMO [9]. To date, several molecular design strategies have been proposed to achieve a small ∆*E*_ST_, such as intramolecular *π*→π* or n→π* charge transfer in a single molecule [10,11,12,13,14,15] and exciplex-based intermolecular charge transfer between an electron-donating molecule and an electron-accepting molecule [16,17,18,19,20].

On the basis of the natural TADF characteristics, the development of exciplex-based TADF emitters for OLEDs has attracted much attention in recent years. Formed between different molecules, the HOMO and LUMO of an exciplex system are naturally located on the electron-donating and electron-accepting molecules, respectively, resulting in almost complete separation and an extremely small Δ*E*_ST_. Since the innovative work of TADF characteristics of exciplexes in 2012 by Adachi et al. [16], a large number of exciplex-based emitters have been developed, whereas a majority of them exhibit green, yellow, or red emission because exciplex formation is usually accompanied by a large red-shift and a broadened structure of the emission spectrum [20]. Accordingly, exciplex-based deep-blue emitters are quite difficult to acquire and in urgent need of exciplex-based full-color displays or white-light OLEDs.

In this study, we developed an efficient deep-blue-emitting exciplex system employing 2,5,8-tris(di(4-fluorophenyl)amine)-1,3,4,6,7,9,9b-heptaazaphenalene (HAP-3FDPA), and 1,3-di(9*H*-carbazol-9-yl)benzene (mCP). HAP-3FDPA, which is composed of a heptazine core and three bis(4-fluorophenyl)amine groups, was designed as an electron acceptor, while mCP possessing two electron-donating carbazole moieties is a widely used host material [21] and was chosen as an electron donor to form exciplex with HAP-3FDPA. Herein, the heptazine core with a planar and rigid heterocyclic system of six C=N bonds surrounding a central sp^2^-hybridised N-atom was chosen as an ideal strong electron-accepting constituent [22,23,24,25,26]. Meanwhile, the three bis(4-fluorophenyl)amine groups were introduced to maintain the electron-withdrawing ability and to increase the solubility of the heptazine derivative. Excitingly, benefitting from the relatively rigid and planar molecular skeletons and strong charge transfer characteristics between HAP-3FDPA and mCP, the OLED incorporating an 8 *wt*% HAP-3FDPA:mCP exciplex system exhibited deep-blue emission with Commission Internationale de l’Eclairage (CIE) coordinates of (0.16, 0.12) and a rather high maximum external quantum efficiency (EQE) of 10.2% along with a fairly low roll-off at high luminance.

## 2. Results and Discussion

**Scheme 1:**
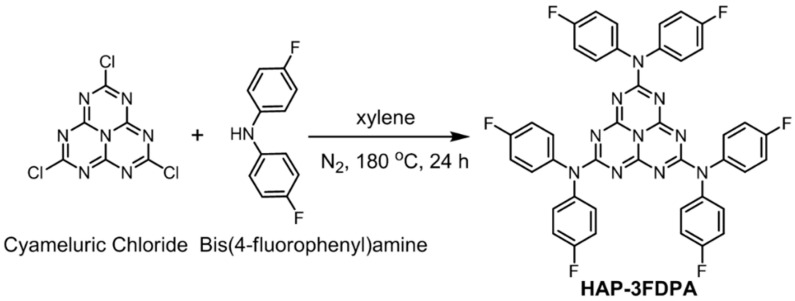
Synthetic route of HAP-3FDPA.

The chemical structure and synthetic route of HAP-3FDPA are depicted in Scheme 1. The target compound of HAP-3FDPA was synthesized by cyameluric chloride and bis(4-fluorophenyl)amine. Thereinto, cyameluric chloride is the key intermediate and was prepared according to the literature [22]. Noteworthily, there is a low yield of 29% for HAP-3FDPA owing to the electron-withdrawing ability of fluorine atoms in bis(4-fluorophenyl)amine. The target compound was characterized and confirmed via ^1^H NMR, ^13^C NMR spectroscopy, and a high-resolution mass spectrometer (HRMS).

**Figure 1:**
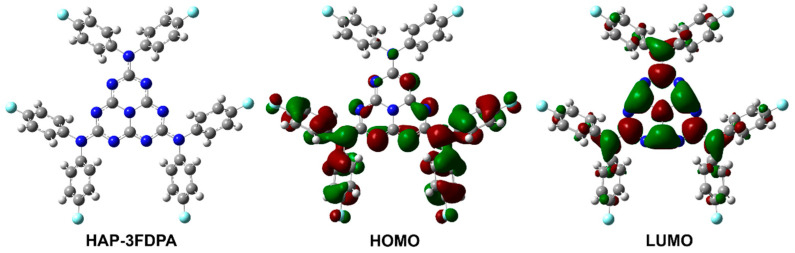
The optimized molecular geometry of the ground state and frontier molecular orbital distributions of HAP-3FDPA by theoretical calculations.

To obtain insight into the electronic structure of HAP-3FDPA, quantum chemical calculations were carried out. The characteristics of molecular configuration, frontier orbitals, and the resulting HOMO and LUMO energy levels were obtained based on density functional theory (DFT) calculation, while the electron transition and excited energy levels of S_1_ and T_1_ were performed by time-dependent density functional theory (TD-DFT) analysis. These results are important to the photophysical properties of HAP-3FDPA and can also provide a theoretical basis for the design of OLED structures. As depicted in Figure 1 and Figure S1 (in Supplementary Materials), the optimized ground state structure revealed that HAP-3FDPA has relatively large dihedral angles of 56–59° between the twisted bis(4-fluorophenyl)amine units and the planar heptazine core, together with that, the HOMO and LUMO are mainly distributed over the bis(4-fluorophenyl)amine units and the heptazine core, respectively. Furthermore, the obvious charge transfer character and the small overlap between the HOMO and LUMO leads to a small Δ*E*_ST_ of 0.29 eV. The calculated HOMO and LUMO levels for HAP-3FDPA are −5.90 and −1.64 eV, respectively. Herein, the deep HOMO and shallow LUMO energy levels should be ascribed to the weak electron-donating ability of bis(4-fluorophenyl)amine units and strong electron-accepting ability of the heptazine core, respectively. Meanwhile, it should be noted that the natural transition orbitals (NTOs) for S_1_ of HAP-3FDPA (HOMO−3 to LUMO) are deriving from localized n→π* transitions involving lone-pair electrons of N heteroatoms and adjacent *π* antibonding molecular orbitals (Figure S2), while the NTOs for T_1_ (HOMO to LUMO or HOMO−1 to LUMO) have the more delocalized *π*→π* transition characters (Figure S3).

**Figure 2:**
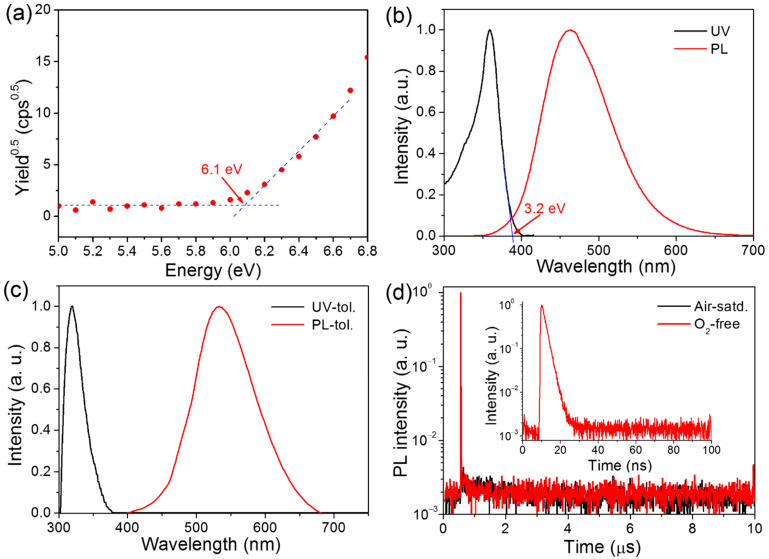
(**a**) The HOMO energy level of HAP-3FDPA determined by atmospheric ultraviolet photoelectron spectroscopy. (**b**) The UV and PL spectra of HAP-3FDPA in a neat film. (**c**) The UV and PL spectra of HAP-3FDPA in diluted toluene (tol.). (**d**) Transient PL decay of HAP-3FDPA in air-saturated (Air-satd.) and oxygen-free (O_2_-free) toluene. Inset: transient PL decay in a time range of 100 ns.

The HOMO energy level was measured by atmospheric ultraviolet photoelectron spectroscopy. As depicted in Figure 2a and Table 1, the HOMO energy level of HAP-3FDPA was determined to be −6.1 eV. As calculated from the HOMO level and the optical energy gap (*E*_g_ = 3.2 eV, Figure 2b), the LUMO energy level was calculated to be −2.9 eV. The ultraviolet-visible (UV) absorption and photoluminescence (PL) spectra of HAP-3FDPA in a neat film are shown in Figure 2b. The intense absorption band with a maximal absorption wavelength (*λ*_abs_) of 268 nm can be assigned to *π*→π* electronic transition in view of the *π*-conjugated molecular system. Meanwhile, HAP-3FDPA in the neat film displayed sky-blue emission with an emission peak wavelength (*λ*_em_) of 464 nm. The UV and PL spectra of HAP-3FDPA in dilute toluene at a concentration of 1 × 10^−4^ mol L^−1^ are shown in Figure 2c. Similar to that of HAP-3FDPA in a neat film, the strong absorption band centered at 319 nm should be attributed to *π*→π* electronic transition. Interestingly, HAP-3FDPA in toluene displayed green emission with *λ*_em_ = 533 nm, indicating that there was a large molecular geometry variation of HAP-3FDPA in a toluene condition in comparison to that in a neat film. Moreover, transient PL decay of HAP-3FDPA both in air-saturated and in oxygen-free toluene was measured (Figure 2d). Apparently, only one prompt component decay could be observed with the lifetime of prompt emission (*τ*_p_) of 1.9 ns. Meanwhile, a quite low PL quantum yield (PLQY) of 5.1% was recorded in both air-saturated and oxygen-free toluene. Consequently, the radiative rate constant of fluorescence (*k*_F_) of HAP-3FDPA was calculated to be 2.7 × 10^7^ s^−1^ according to the equation of *k*_F_ = PLQY/*τ*_p_. Thus, the oxygen-independent transient PL decay and PLQY indicate the absence of delayed fluorescence, which probably originated from the whole electron-withdrawing molecular structure of HAP-3FDPA.

**Figure 3:**
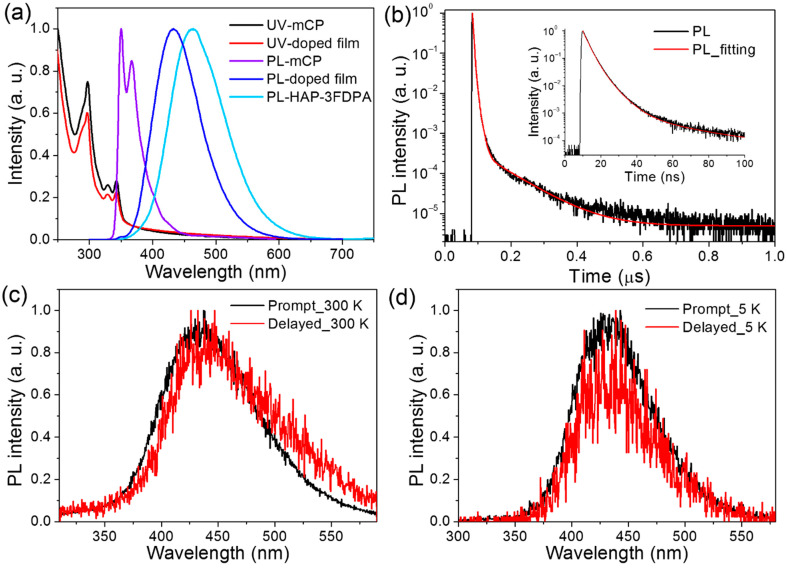
(**a**) The UV and PL spectra of mCP, HAP-3FDPA in neat film, and 8 *wt*% HAP-3FDPA:mCP-doped film at 300 K. (**b**) Transient PL decay of the 8 *wt*% HAP-3FDPA:mCP-doped film at 300 K. Inset: transient PL decay with a time range of 100 ns. (**c**) Prompt and delayed PL spectra of the 8 *wt*% HAP-3FDPA:mCP-doped film at 300 K. (**d**) Prompt and delayed PL spectra of the 8 *wt*% HAP-3FDPA:mCP-doped film at 5 K.

To verify that HAP-3FDPA can be used as an electron acceptor in the exciplex system, 1,3-di(9*H*-carbazol-9-yl)benzene (mCP) possessing two electron-donating carbazole moieties and a high T_1_ level is a popular host material and was chosen as the electron donor to form exciplex with HAP-3FDPA [21]. Subsequently, an 8 *wt*% HAP-3FDPA:mCP-doped film was fabricated and characterized, and the photophysical properties are exhibited in Figure 3 and Table 1. Herein, the concentration of 8 *wt*% was chosen based on the optimization of luminescence efficiencies at various concentrations (Table S1). Most strikingly, the 8 *wt*% HAP-3FDPA:mCP-doped film showed deep-blue emission with *λ*_em_ = 433 nm (Figure 3a), which is significantly blue-shifted compared to those in a neat film (464 nm) and toluene (533 nm). Meanwhile, the structureless and smooth emission spectrum presented a narrow full width at half maximum (FWHM) of 87 nm in comparison with those of HAP-3FDPA in a neat film (101 nm) and in diluted toluene (102 nm), which is beneficial to the improvement of color purity for exciplex systems. As we know, there is usually a red-shifted and broadened emission spectrum for exciplex systems compared to those of corresponding single compounds [27,28,29,30,31,32]. Therefore, a large number of green- and red-emitting exciplex systems have been developed, whereas deep-blue-emitting exciplex systems are quite difficult to realize [20]. Hence, the PL spectrum of the 8 *wt*% HAP-3FDPA:mCP-doped film is remarkably different from the emission tendency of traditional exciplex systems.

**Scheme 2:**
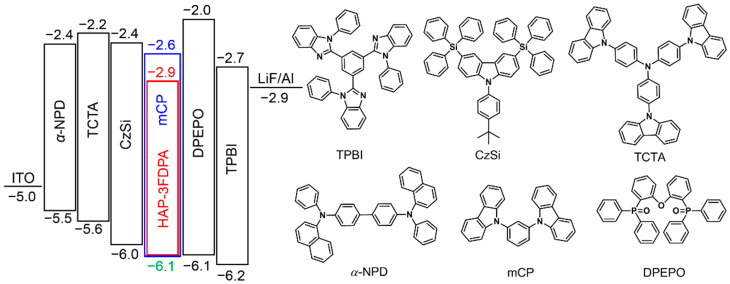
The energy diagram of the HOMO and LUMO levels of materials utilized in the OLED device and corresponding chemical molecular structures.

To confirm the exciplex emission of the 8 *wt*% HAP-3FDPA:mCP-doped film, transient PL decay of the doped film at 300 K was measured and is shown in Figure 3b and Figure S4. Obviously, the decay process can be divided into prompt and delayed components. To better elucidate the exciplex emission, prompt and delayed emission spectra of the 8 *wt*% HAP-3FDPA:mCP-doped film at both 300 and 5 K were characterized (Figure 3c,d). The well-overlapped prompt and delayed emission spectra at 300 K manifest that all photons are generated from the same excited state. Meanwhile, the considerably good overlap of fluorescence and phosphorescence spectra at 5 K confirms that the doped film possesses an extremely small Δ*E*_ST_. Thus, the 8 *wt*% HAP-3FDPA:mCP-doped film can be considered as an exciplex system. Additionally, the strong prompt component with *τ*_p_ = 3.0 ns should be assigned to exciplex-based fluorescence, while the weak delayed component with two comparably short lifetimes (*τ*_d_) of 8.0 and 86.1 ns can be attributed to the exciplex-based delayed fluorescence. It is noteworthy that the 8 *wt*% HAP-3FDPA:mCP exciplex system displayed a relatively high PLQY of 53.2%, which is much higher than that of HAP-3FDPA in dilute toluene (5.1%), implying efficient radiative transition of singlet excitons from S_1_ to the ground state. Furthermore, to elucidate the mechanism of exciplex emission, as shown in Figure S5, the HOMO and LUMO levels of mCP were measured to be −6.1 and −2.6 eV by atmospheric ultraviolet photoelectron spectroscopy and UV spectra, respectively. Obviously, the aforementioned deep-blue emission of the 8 *wt*% HAP-3FDPA:mCP exciplex system should be attributed to the shallow LUMO (−2.9 eV) of HAP-3FDPA and deep HOMO (−6.1 eV) of mCP, leading to a large energy gap of 3.2 eV for the exciplex formation [16]. Meanwhile, the narrow FWHM may stem from the fairly rigid and planar geometries of HAP-3FDPA and mCP, which tend to result in tight molecular packing and strong intermolecular interactions [18,33]. Additionally, blend films of 8 *wt*% HAP-3FDPA:DPEPO (bis(2-(diphenylphosphino)phenyl) ether oxide) and 8 *wt*% HAP-3FDPA:TCTA (4,4′,4″-tris(*N*-carbazolyl)triphenylamine) were fabricated and compared with that of 8 *wt*% HAP-3FDPA:mCP-doped film. As shown in Figures S6 and S7, no delayed component was observed in the transient PL decay of 8 *wt*% HAP-3FDPA:DPEPO blend film, indicating the absence of exciplex emission due to the electron-accepting character of the DPEPO molecule. Meanwhile, the PL spectrum of the 8 *wt*% HAP-3FDPA:TCTA blend film showed an apparent red-shift as compared to that of the 8 *wt*% HAP-3FDPA:mCP-doped film, which should be ascribed to the shallower HOMO of TCTA than that of mCP (Scheme 2).

**Figure 4:**
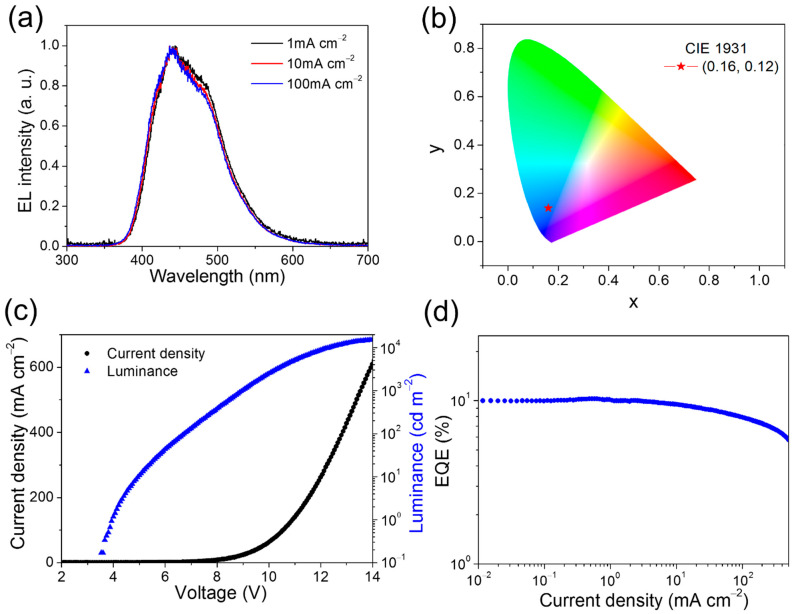
EL characteristics of the 8 *wt*% HAP-3FDPA:mCP exciplex-based OLED. (**a**) The EL spectra at various current densities. (**b**) The CIE coordinates of (0.16, 0.12) for EL spectra. (**c**) The current efficiency-voltage-luminance (*J*-*V*-*L*) characteristics. (**d**) EQE as a function of current density.

To evaluate EL performance of the 8 *wt*% HAP-3FDPA:mCP exciplex system, an OLED device incorporating an emitting layer of the exciplex system was fabricated with a structure of ITO (indium tin oxide)/α-NPD (*N*,*N*′-diphenyl-*N*,*N*′-bis(1-naphthyl)-1,10-biphenyl-4,4′-diamine) (30 nm)/TCTA (20 nm)/CzSi (9-(4-*tert*-butylphenyl)-3,6-bis(triphenylsilyl)-9H-carbazole) (10 nm)/ 8 *wt*% HAP-3FDPA:mCP (20 nm)/DPEPO (10 nm)/TPBI (1,3,5-tris(*N*-phenylbenzimidazol-2-yl)benzene) (30 nm)/LiF (1 nm)/Al (100 nm). The energy diagram and chemical molecular structures of organic compounds employed in the OLED device are depicted in Scheme 2. The EL spectra of this device measured at 1, 10, 100 mA cm^−2^ are well-overlapped with a maximum EL peak (*λ*_EL_) of 437 nm and are similar to the PL spectrum of the emitting layer (Figure 4a). Meanwhile, the photo energy of the exciplex was calculated to be 3.2 eV from the onset of the EL spectrum (373 nm), which is in good agreement with the energy difference between the LUMO of HAP-3FDPA and the HOMO of mCP (Figure S5). More importantly, the OLED showed deep-blue emission with CIE coordinates of (0.16, 0.12), a turn-on voltage (*V*_on_) of 4.0 V, a peak luminance (*L*_max_) of 15,148 cd m^−2^, and a rather high maximum external quantum efficiency (EQE) of 10.2% without any light out-coupling enhancement (Figure 4b–d and Table 2). Moreover, it is noteworthy that there is a rather low-efficiency roll-off at high luminance for the exciplex-based OLED, with 10.0%, 9.0%, 7.7%, and 6.7% at 100, 1000, 5000, and 10,000 cd m^−2^, respectively. The excellent EL performance of the OLED employing an 8 *wt*% HAP-3FDPA:mCP exciplex system should be predominantly ascribed to efficient up-conversion of triplet excitons from T_1_ to S_1_ through the TADF process under electrical excitation.

## 3. Materials and Methods

### 3.1. Synthesis of 2,5,8-Tris(di(4-fluorophenyl)amine)-1,3,4,6,7,9,9b-heptaazaphenalene (HAP-3FDPA)

A flame-dried Schlenk tube with a magnetic stir bar was charged with mixture of cyameluric chloride (1.09 mmol, 300 mg), bis(4-fluorophenyl)amine (17.7 mmol, 3.6 g) and dry xylene (20 mL) under an N_2_ atmosphere. The resulting mixture was heated at 180 °C for 24 h. After cooling to room temperature, the solvent was removed by vacuum distillation. The residue was purified by column chromatography on silica gel and recrystallized from ethyl acetate/petroleum ether mixtures to provide the desired product as white solid (250 mg, 29%). ^1^H NMR (400 MHz, DMSO-*d*_6_): *δ* (ppm) = 7.28–7.34 (m, 1H), 7.17 (t, *J* = 8.8 Hz, 1H). ^13^C NMR (100 MHz, DMSO-*d*_6_): *δ* (ppm) = 164.38, 161.67, 159.23, 155.81, 139.22, 130.02, 129.94, 116.22, 115.99. HRMS (ESI^+^): calcd. for C_42_H_25_F_6_N_10_ [M+H]^+^ 783.2090, found 783.2092.

### 3.2. OLED Fabrication and Measurement

The OLED was fabricated by vacuum thermal evaporation under pressure lower than 5 × 10^−4^ Pa. A 150 nm-thick indium-tin-oxide (ITO) precoated glass substrate was used as the anode. Prior to the deposition of the organic layers and cathode, the substrate was firstly cleaned with ultra-purified water, acetone, and isopropyl alcohol (IPA) in sequence, then treated with UV-ozone for 15 min and finally transferred to a vacuum thermal deposition system. The intersection of ITO and the metal electrodes gave an active device area of 4 mm^2^. The OLED device was characterized under atmospheric conditions without any encapsulation or light out-coupling enhancement. The EL spetrum, EQE, and current density–voltage–luminance (*J*–*V*–*L*) characteristics of the OLED were recorded with a semiconductor parameter analyzer (E5270, Agilent, Santa Clara, CA, USA) and an optical power meter (1930C, Newport, Irvine, CA, USA). EL spectra were recorded using a multi-channel spectrometer (SD2000, Ocean Optics, Dunedin, FL, USA).

## 4. Conclusions

In summary, we designed an efficient exciplex-based deep-blue emitter incorporating 2,5,8-tris(di(4-fluorophenyl)amine)-1,3,4,6,7,9,9b-heptaazaphenalene (HAP-3FDPA) as the electron acceptor and 1,3-di(9*H*-carbazol-9-yl)benzene (mCP) as the electron donor. The 8 *wt*% HAP-3FDPA:mCP exciplex system exhibited deep-blue emission with *λ*_em_ = 433 nm and a fairly small Δ*E*_ST_, giving rise to efficient exciton up-conversion and a high PLQY of 53.2%. More importantly, an OLED containing this exciplex system as an emitting layer showed deep-blue emission with CIE coordinates of (0.16, 0.12), a peak luminance (*L*_max_) of 15148 cd m^−2^, and a rather high maximum external quantum efficiency (EQE) of 10.2% along with a fairly low roll-off at high luminance. These findings are of fundamental interest for the development of deep-blue OLEDs based on exciplex systems. Through the elaborate molecular design of electron donors and acceptors, we believe that highly efficient exciplex-based deep-blue OLEDs can be expected.

## Figures and Tables

**Table 1:** The photophysical properties of HAP-3FDPA and an 8 *wt*% HAP-3FDPA:mCP-doped film.

Compound	HOMO/LUMO (eV)	*λ*_abs_ (nm)	*λ*_em_ (nm)	*τ*_p_ (ns)	*τ*_d_ (ns)	PLQY	FWHM (nm)
HAP-3FDPA	−6.1/−2.9	359 ^a^/319 ^b^	464 ^a^/533 ^b^	1.9 ^b^	-	5.1% ^b^	101 ^a^/102 ^b^
Doped film	-	342	433	3.0	8.0, 86.1	53.2%	87

^a^ Measured in a neat film; ^b^ measured in oxygen-free toluene.

**Table 2:** The OLED performance based on an 8 *wt*% HAP-3FDPA:mCP exciplex system.

Emitter	*V*_on_ (V) ^a^	*λ*_EL_ (nm)	*L*_max_ (cd m^−2^)	EQE (%) ^b^	CIE (x, y)
8 *wt*% HAP-3FDPA:mCP	4.0	437	15,148	10.2/10.0/9.0/7.7/6.7	0.16, 0.12

^a^ Turn-on voltage at 1 cd m^−2^, ^b^ the maximum EQE, EQEs at 100, 1000, 5000, and 10,000 cd m^−2^, respectively.

## Data Availability

The data presented in this study are available on request from the corresponding author.

## Supplementary Materials

Instrumentation; Quantum chemical calculations; Figure S1: Frontier molecular orbital distributions and energy levels of the lowest excited singlet and triplet states of HAP-3FDPA by theoretical calculations; Figure S2: The natural transition orbitals (197→201) for the lowest excited singlet state (S_1_) of HAP-3FDPA by theoretical calculations; Figure S3: The natural transition orbitals (199→201) and (200→201) for the lowest excited triplet state (T_1_) of HAP-3FDPA by theoretical calculations; Figure S4: The transient PL decay image of 8 *wt*% HAP-3FDPA:mCP-doped film in the time range of 2 μs; Figure S5: (a) The HOMO energy level of mCP determined by atmospheric ultraviolet photoelectron spectroscopy. (b) The UV spectra of mCP in a neat film. The optical energy gap of mCP was calculated to be 3.5 eV. Therefore, the LUMO energy level of mCP could be calculated to be −2.6 eV; Figure S6: The transient PL decay of 8 *wt*% HAP-3FDPA:DPEPO-doped film in the time range of 5 μs; Figure S7: The PL spectrum of 8 *wt*% HAP-3FDPA:TCTA-doped film as compared to that of 8 *wt*% HAP-3FDPA:mCP; Figure S8: The energy diagram of the 8 *wt*% HAP-3FDPA:mCP exciplex system; Figure S9: ^1^H NMR spectrum of HAP-3FDPA in DMSO-*d*_6_; Figure S10: ^13^C NMR spectrum of HAP-3FDPA in DMSO-*d*_6_; Table S1: The PLQYs of HAP-3FDPA:mCP doped films at various concentrations.
