# Supplementary material for: Efficient Exciplex-Based Deep-Blue Organic Light-Emitting Diodes Employing a Bis(4-fluorophenyl)amine-Substituted Heptazine Acceptor
Source: Molecules. 2021 Sep 13;26(18):5568. doi: 10.3390/molecules26185568 (PMC8466596; doi:10.3390/molecules26185568)
Supplement: Supplementary file 1 [file molecules-26-05568-s001.zip › molecules-1333185-supplementary.pdf]

Supplementary Materials

Efficient Exciplex-Based Deep-Blue Organic Light-Emitting Diodes Employing a Bis(4-fluorophenyl) amine-Subsitituted Heptazine Acceptor

Jie Li [1], Heqi Gong [1], Jincheng Zhang [1], Hui Liu [1], Li Tao [1], Yanqing Wang [2] and Qiang Guo [1,*]

[1]*College of Optoelectronic Engineering, Chengdu University of Information Technology, Chengdu 610225, China*

[2]*College of Polymer Science and Engineering, Sichuan University, Chengdu 610065, China*

**Correspondence: qiangguo@cuit.edu.cn*

Table of Contents

1. Instrumentation

2. Quantum Chemical Calculations

3. Photophysical Properties

4. ^{1}H and ^{13}C NMR spectra of HAP-3FDPA

5. References

1. Instrumentation

NMR spectra were obtained on a Varian Inova 400 spectrometer. The ^{1}H NMR (400 MHz) and ^{13}C NMR (100 MHz) chemical shifts were measured relative to DMSO-d_6 as the internal reference (DMSO-d_6: δ = 2.50 ppm for ^{1}H and δ = 39.52 ppm for ^{13}C respectively). High-resolution mass spectra (HRMS) were obtained with Waters-Q-TOF-Premier (ESI^{+}). The UV and PL spectra were recorded with a Shimadzu UV-2550 spectrophotometer and Shimadzu RF-5301PC fluorescence spectrometer, respectively. The PLQY and transient PL decay were recorded using a Hamamatsu C9920-02 and a Hamamatsu C11367-03 measurement system, respectively. Transient PL decay characteristics of film samples under vacuum conditions were measured on a streak camera (C4334, Hamamatsu Photonics) equipped with cryostat using a YAG laser. The highest occupied molecular orbital (HOMO) energy levels of HAP-3FDPA and mCP were determined by atmospheric ultraviolet photoelectron spectroscopy (AC-3E, Riken Keiki), and the corrsponding lowest unoccupied molecular orbital (LUMO) levels were calculated by subtracting the energy gaps from the HOMO levels. Oxygen-free sample solutions (1×10^{-4} mol L^{-1}) were degassed with N_2 for 15 min prior to use unless otherwise indicated. Pure and doped films were deposited on quartz and silicon substrates by vacuum thermal evaporation at a pressure lower than 5×10^{-4} Pa.

2. Quantum Chemical Calculations

All calculations were performed using the Gaussian 09 program package. The HOMO and LUMO of HAP-3FDPA were calculated using the nonlocal density functional of Becke's 3-parameters employing Lee-Yang-Parr functional (B3LYP) with the 6-31G(d) basis set. Their S_1 and T_1 were calculated by the time-dependent density functional theory (TD-DFT) method at the optimized ground-state geometries using the B3LYP mode with a 6-31G(d) basis set [S1].

2.1 The Optimized Geometry Data for HAP-3FDPA (unit: Å)

C	-7.01210465	-1.70088527	-0.09624241
C	-6.54392461	-2.42884946	0.99099161
C	-5.18769035	-2.74392982	1.05169564
C	-4.31985395	-2.33820662	0.03287356
C	-4.81732526	-1.61455175	-1.05631107
C	-6.16925764	-1.28681791	-1.12185208
N	-2.93472063	-2.72478931	0.09912647
C	-1.92679378	-1.79004971	0.06266245
N	-2.30715943	-0.49936494	0.06483414
C	-1.34478238	0.41434050	0.02839837
N	0.00081352	-0.00108659	-0.00452858
C	0.31406057	-1.37413160	-0.00100236

N	-0.66886392	-2.26584232	0.02838510
N	-1.62512233	1.71193362	0.02946096
C	-0.58385735	2.56328517	-0.00495091
N	0.72343172	2.24719198	-0.04221759
C	1.03337410	0.95637302	-0.04056061
N	2.29677222	0.55019137	-0.07752022
C	2.51368560	-0.77765666	-0.06913366
N	1.58637309	-1.75152253	-0.02736657
N	-0.88960003	3.90391712	0.00001975
N	3.82691654	-1.18377219	-0.10528852
C	4.18567442	-2.57485716	-0.19601790
C	-2.25125892	4.36642864	-0.06201825
C	0.13570589	4.91146572	0.07147079
C	0.17934428	5.90659226	-0.91033295
C	1.12686065	6.92631973	-0.84109708
C	2.02792346	6.92975838	0.21672683
C	2.00453309	5.95186607	1.20515365
C	1.04784761	4.94250341	1.13213814
C	5.06032418	-3.11228057	0.75410718
C	5.46674172	-4.44259541	0.66802628
C	4.98153466	-5.22290662	-0.37438456
C	4.11195608	-4.71167451	-1.33153914
C	3.71947132	-3.37843805	-1.24258944
C	-2.72146608	5.22751784	0.93470183
C	-4.01875562	5.73336662	0.87659541
C	-4.83381111	5.36056633	-0.18536947
C	-4.38922116	4.50651090	-1.18870822
C	-3.08817211	4.01361846	-1.12642164
C	-2.65736913	-4.13314665	0.20473396
C	-1.88947322	-4.64537068	1.25672480
C	-1.66707844	-6.01622747	1.36004503
C	-2.23044581	-6.86330705	0.41207618
C	-3.00539187	-6.38035978	-0.63537029
C	-3.21385017	-5.00603874	-0.73602622
F	-8.32315558	-1.39068935	-0.16174329
F	-2.02204708	-8.19209959	0.51488909
F	-6.09129465	5.84454633	-0.24751864
F	5.36837973	-6.51215519	-0.46302700
F	2.94908957	7.91236184	0.28992221
C	4.90919046	-0.23596968	-0.05527479
C	5.86730127	-0.24648730	-1.07405501
C	6.95483868	0.62383891	-1.02984155
C	7.06304638	1.50315745	0.04075836
C	6.12371903	1.53255441	1.06570800
C	5.04551476	0.65238141	1.01704185

F	8.11195924	2.34974584	0.09027066
H	-7.23240564	-2.73364195	1.77192129
H	-4.79930230	-3.31137400	1.89137647
H	-4.14295479	-1.29705541	-1.84307353
H	-6.57473701	-0.72186797	-1.95444495
H	-0.53395358	5.88351415	-1.72798066
H	1.17654925	7.70587354	-1.59383376
H	2.72408632	5.99140662	2.01601183
H	1.01704570	4.16893257	1.89046405
H	5.42705649	-2.48482274	1.56014245
H	6.14390603	-4.87656476	1.39600747
H	3.75599282	-5.35283854	-2.13089287
H	3.03726748	-2.96480002	-1.97573888
H	-2.06734996	5.50472066	1.75517260
H	-4.40150230	6.40140199	1.64080597
H	-5.05617951	4.24114400	-2.00207410
H	-2.72484995	3.34235076	-1.89576933
H	-1.45296575	-3.97024444	1.98308750
H	-1.06904595	-6.43199335	2.16395656
H	-3.42867850	-7.07191512	-1.35608463
H	-3.81575089	-4.60759734	-1.54642550
H	5.76216767	-0.94167051	-1.90084521
H	7.70741250	0.63111340	-1.81109406
H	6.24540180	2.23291318	1.88515984
H	4.29993582	0.66339222	1.80349778

2.2 Excitation Energies and Oscillator Strengths for HAP-3FDPA

Excited State 1: Triplet-A 3.2734 eV 378.77 nm f=0.0000

198 → 202 0.11849
198 → 203 0.10932
199 → 201 0.42667
199 → 203 -0.11350
200 → 201 0.45364
200 → 202 -0.12793

Excited State 2: Triplet-A 3.2746 eV 378.62 nm f=0.0000

198 → 202 0.10563
198 → 203 -0.12371
199 → 201 0.45332
199 → 202 0.13109
200 → 201 -0.42716
200 → 203 -0.10262

Excited State 3: Triplet-A 3.3514 eV 369.95 nm f=0.0000

198 → 201 0.57236
199 → 202 0.21785

200 → 203 0.23633
200 → 213 0.10649
Excited State 4: Triplet-A 3.5277 eV 351.46 nm f=0.0000
197 → 201 0.55171
198 → 208 -0.15420
199 → 203 -0.14323
199 → 209 0.16301
200 → 202 0.14113
200 → 207 0.15575
Excited State 5: Singlet-A 3.5639 eV 347.89 nm f=0.0000
197 → 201 0.67884
Excited State 6: Singlet-A 3.6477 eV 339.89 nm f=0.3930
200 → 201 0.67448
Excited State 7: Singlet-A 3.6811 eV 336.81 nm f=0.4048
199 → 201 0.67851
Excited State 8: Singlet-A 3.6876 eV 336.22 nm f=0.0009
198 → 201 0.64391

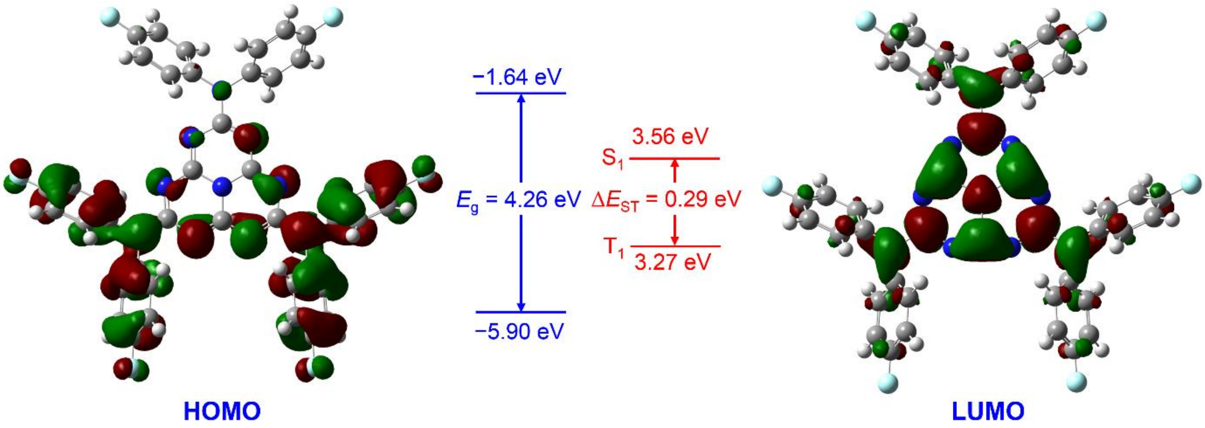

Figure S1. Frontier molecular orbital distributions and energy levels of the lowest excited singlet and triplet states of HAP-3FDPA by theoretical calculations.

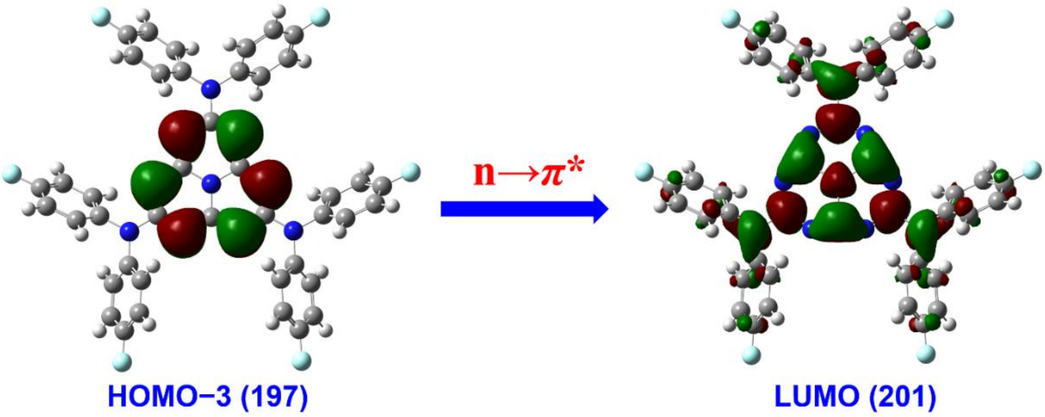

Figure S2. The natural transition orbitals (197 → 201) for the lowest excited singlet state (S_1) of HAP-3FDPA by theoretical calculations.

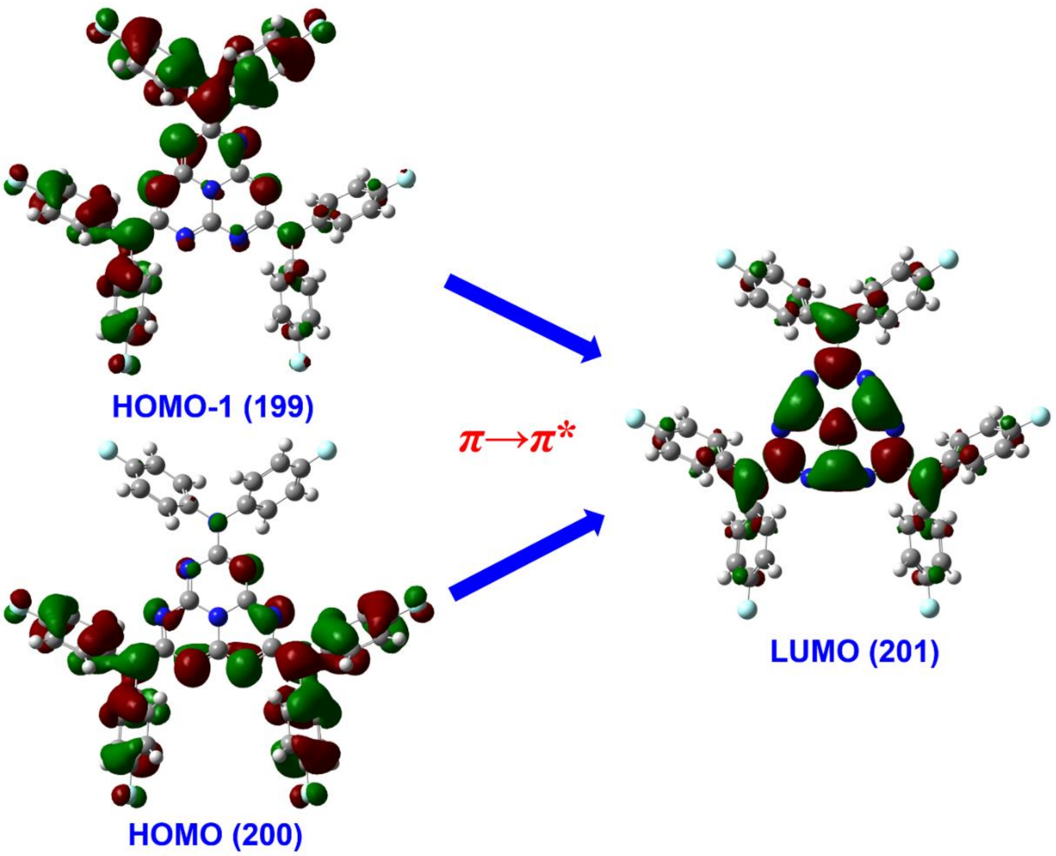

Figure S3. The natural transition orbitals (199 → 201) and (200→ 201) for the lowest excited triplet state (T_1) of HAP-3FDPA by theoretical calculations.

3. Photophysical Properties

Table S1. The PLQYs of HAP-3FDPA:mCP doped films at various concentrations.

Concentration	PLQY
8 wt% HAP-3FDPA:mCP	53.2%
25 wt% HAP-3FDPA:mCP	41.6%
50 wt% HAP-3FDPA:mCP	37.1%
100 wt% HAP-3FDPA:mCP	5.1%

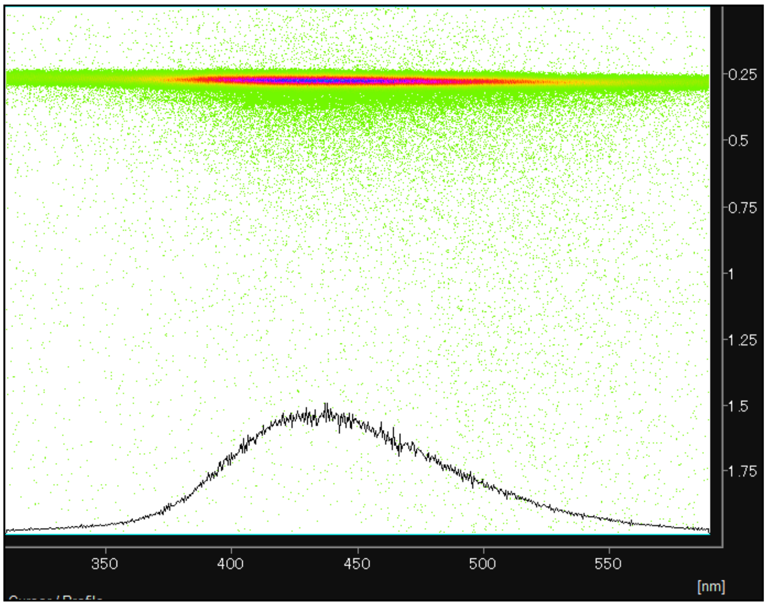

Figure S4. The transient PL decay image of 8 wt% HAP-3FDPA:mCP doped film in the time range of 2 μs.

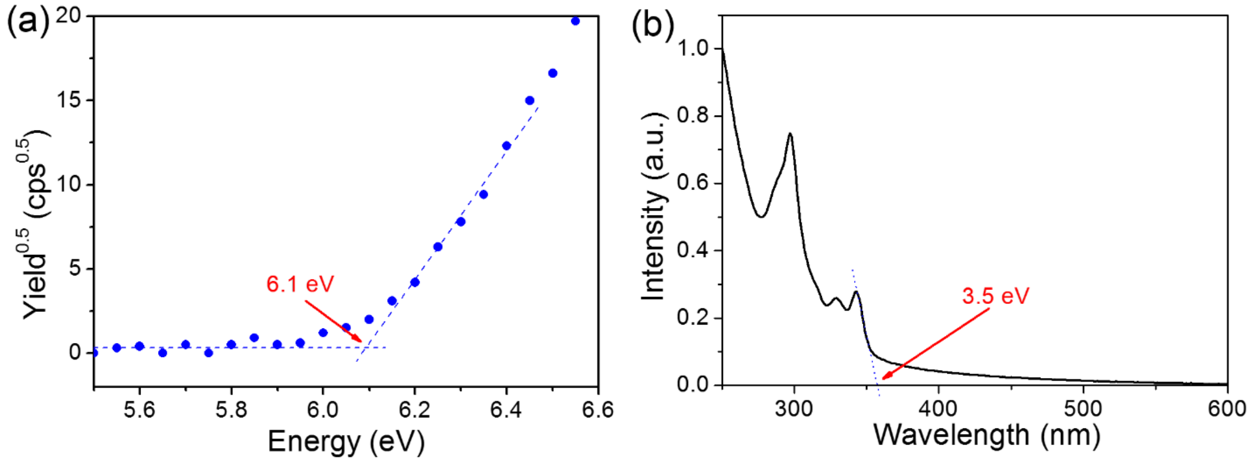

Figure S5. (a) The HOMO energy level of mCP determined by atmospheric ultraviolet photoelectron spectroscopy. **(b)** The UV spectra of mCP in a neat film. The optical energy gap of mCP was calculated to be 3.5 eV. Therefore, the LUMO energy level of mCP could be calculated to be −2.6 eV.

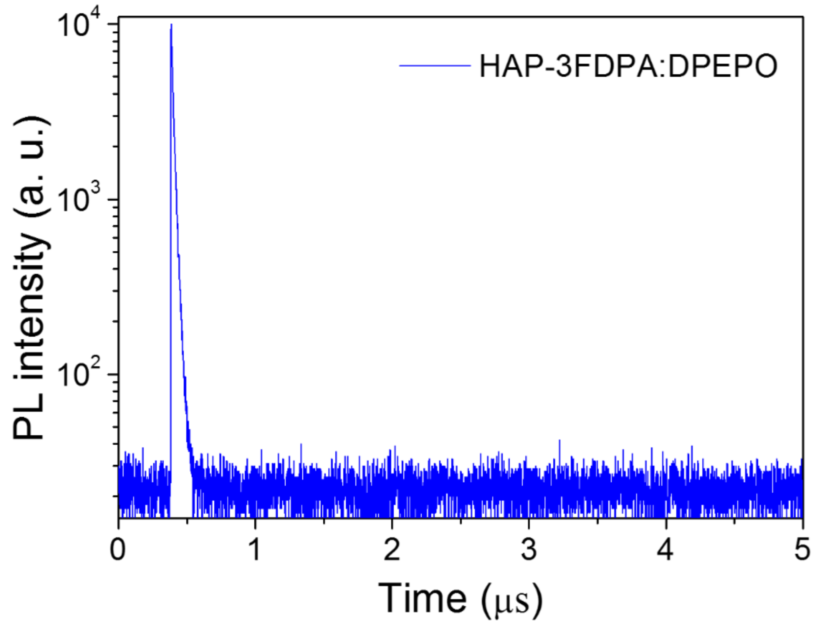

Figure S6. The transient PL decay of 8 wt% HAP-3FDPA:DPEPO doped film in the time range of 5 μs.

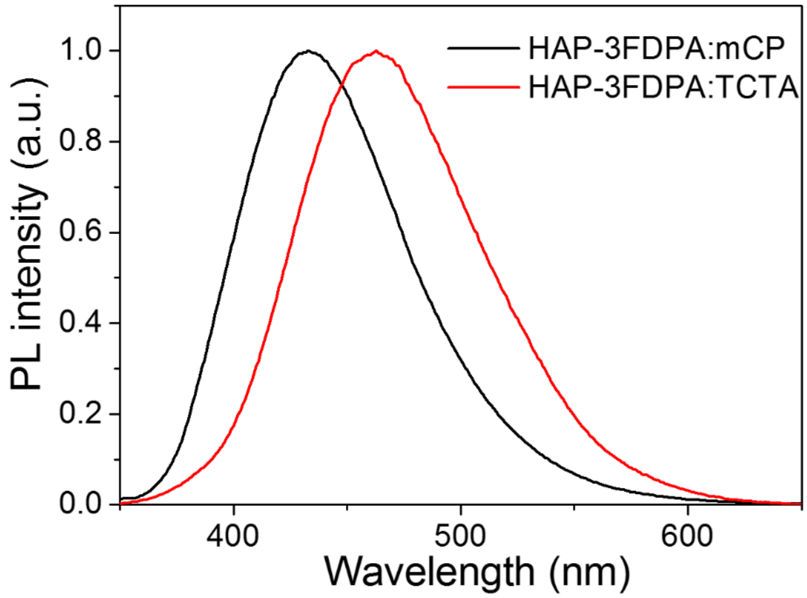

Figure S7. The PL spectrum of 8 wt% HAP-3FDPA:TCTA doped film as compared to that of 8 wt% HAP-3FDPA:mCP.

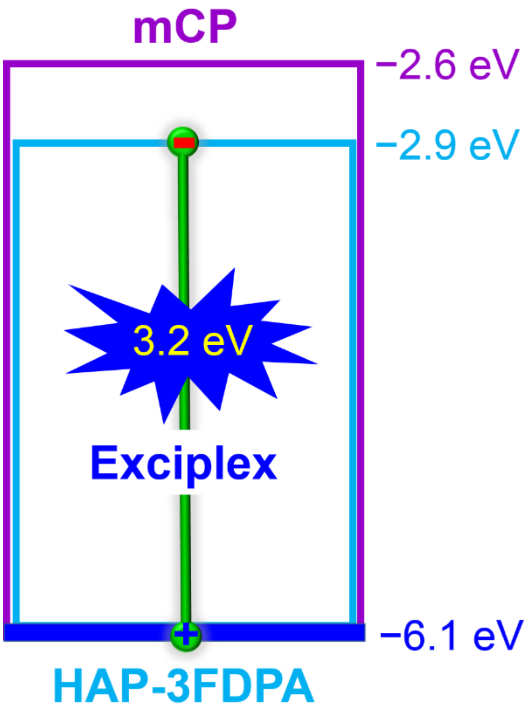

Figure S8. The energy diagram of the 8 wt% HAP-3FDPA:mCP exciplex system.

4. ^{1}H and ^{13}C NMR spectra of HAP-3FDPA

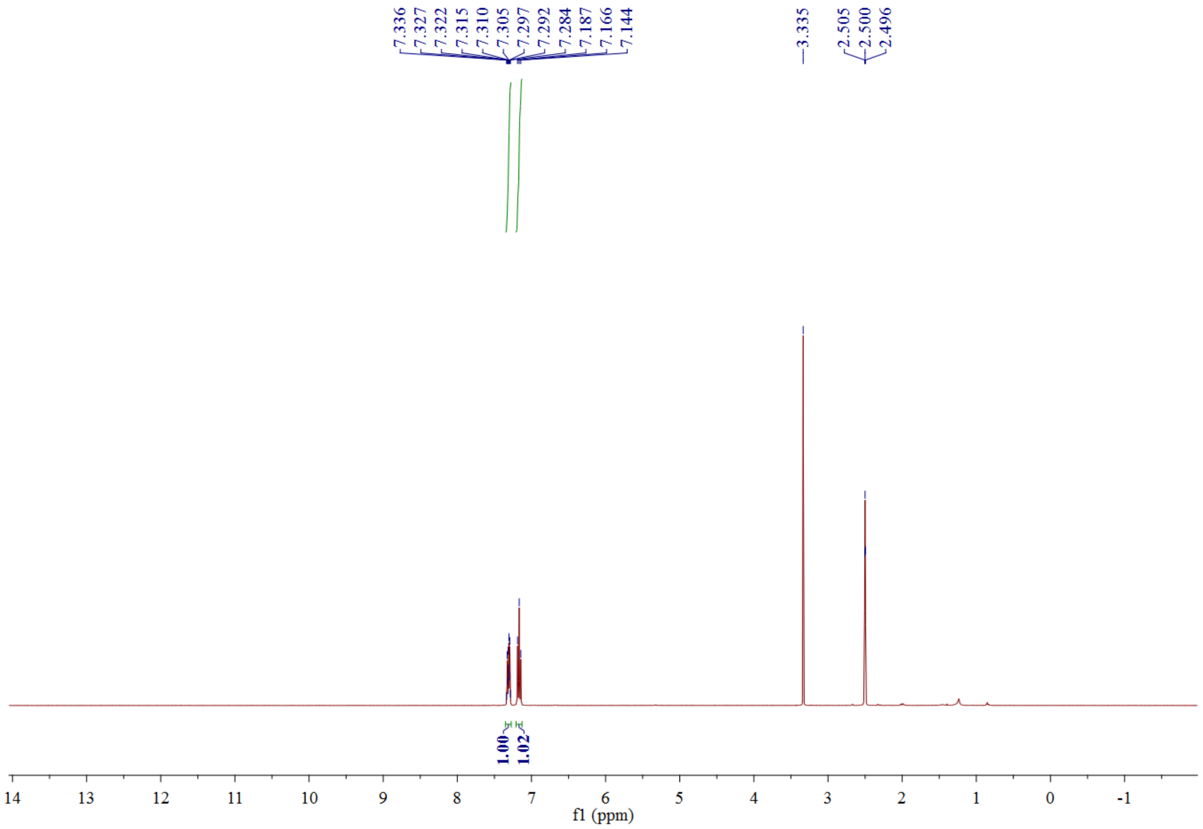

Figure S9. ^{1}H NMR spectrum of HAP-3FDPA in DMSO-d_6.

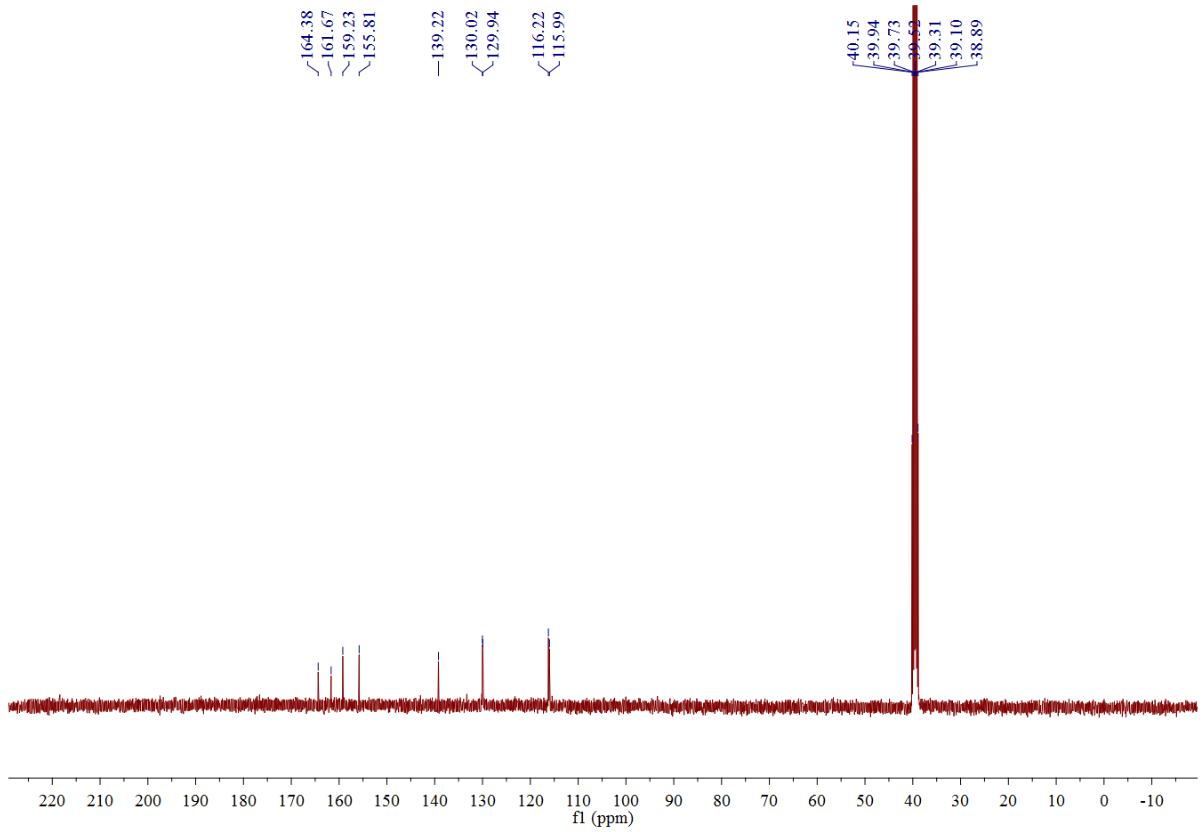

Figure S10. ^{13}C NMR spectrum of HAP-3FDPA in DMSO-d_6.

5. References

S1 M. J. Frisch, G. W. Trucks, H. B. Schlegel, G. E. Scuseria, M. A. Robb, J. R. Cheeseman, G. Scalmani, V. Barone, B. Mennucci, G. A. Petersson, H. Nakatsuji, M. Caricato, X. Li, H. P. Hratchian, A. F. Izmaylov, J. Bloino, G. Zheng, J. L. Sonnenberg, M. Hada, M. Ehara, K. Toyota, R. Fukuda, J. Hasegawa, M. Ishida, T. Nakajima, Y. Honda, O. Kitao, H. Nakai, T. Vreven, J. A. Montgomery, Jr., J. E. Peralta, F. Ogliaro, M. Bearpark, J. J. Heyd, E. Brothers, K. N. Kudin, V. N. Staroverov, R. Kobayashi, J. Normand, K. Raghavachari, A. Rendell, J. C. Burant, S. S. Iyengar, J. Tomasi, M. Cossi, N. Rega, J. M. Millam, M. Klene, J. E. Knox, J. B. Cross, V. Bakken, C. Adamo, J. Jaramillo, R. Gomperts, R. E. Stratmann, O. Yazyev, A. J. Austin, R. Cammi, C. Pomelli, J. W. Ochterski, R. L. Martin, K. Morokuma, V. G. Zakrzewski, G. A. Voth, P. Salvador, J. J. Dannenberg, S. Dapprich, A. D. Daniels, Ö. Farkas, J. B. Foresman, J. V. Ortiz, J. Cioslowski, and D. J. Fox, *Gaussian 09, Revision C.01*. (Gaussian, Inc., 2009).
